# Genomics of parallel adaptation at two timescales in *Drosophila*

**DOI:** 10.1371/journal.pgen.1007016

**Published:** 2017-10-02

**Authors:** Li Zhao, David J. Begun

**Affiliations:** 1 Department of Evolution and Ecology, University of California Davis, Davis, California, United States of America; 2 Laboratory of Evolutionary Genetics and Genomics, The Rockefeller University, New York, New York, United States of America; University of Arizona, UNITED STATES

## Abstract

Two interesting unanswered questions are the extent to which both the broad patterns and genetic details of adaptive divergence are repeatable across species, and the timescales over which parallel adaptation may be observed. *Drosophila melanogaster* is a key model system for population and evolutionary genomics. Findings from genetics and genomics suggest that recent adaptation to latitudinal environmental variation (on the timescale of hundreds or thousands of years) associated with Out-of-Africa colonization plays an important role in maintaining biological variation in the species. Additionally, studies of interspecific differences between *D*. *melanogaster* and its sister species *D*. *simulans* have revealed that a substantial proportion of proteins and amino acid residues exhibit adaptive divergence on a roughly few million years long timescale. Here we use population genomic approaches to attack the problem of parallelism between *D*. *melanogaster* and a highly diverged conger, *D*. *hydei*, on two timescales. *D*. *hydei*, a member of the *repleta* group of *Drosophila*, is similar to *D*. *melanogaster*, in that it too appears to be a recently cosmopolitan species and recent colonizer of high latitude environments. We observed parallelism both for genes exhibiting latitudinal allele frequency differentiation within species and for genes exhibiting recurrent adaptive protein divergence between species. Greater parallelism was observed for long-term adaptive protein evolution and this parallelism includes not only the specific genes/proteins that exhibit adaptive evolution, but extends even to the magnitudes of the selective effects on interspecific protein differences. Thus, despite the roughly 50 million years of time separating *D*. *melanogaster* and *D*. *hydei*, and despite their considerably divergent biology, they exhibit substantial parallelism, suggesting the existence of a fundamental predictability of adaptive evolution in the genus.

## Author summary

Both local adaptation on short timescales and the long-term accumulation of adaptive differences between species have recently been investigated using comparative genomic and population genomic approaches in several species. However, the repeatability of adaptive evolution at the genetic level is poorly understood. Here we attack this problem by comparing patterns of long and short-term adaptation in *Drosophila melanogaster* to patterns of adaptation on two timescales in a highly diverged congener, *Drosophila hydei*. We found, despite the fact that these species diverged from a common ancestor roughly 50 million years ago, the population genomics of latitudinal allele frequency differentiation shows that there is a substantial shared set of genes likely playing a role in the short term adaptive divergence of populations in both species. Analyses of longer-term adaptive protein divergence for the *D*. *hydei*-*D*. *mojavensis* and *D*. *melanogaster*-*D*. *simulans* clades reveal a striking level of parallel adaptation. This parallelism includes not only the specific genes/proteins that exhibit adaptive evolution, but extends even to the magnitudes of the selective effects on interspecific protein differences.

## Introduction

While parallel phenotypic evolution has long been recognized as one of the strongest pieces of evidence for adaptation [1], the general repeatability of adaptive evolution in natural populations remains poorly understood. In large part this is because only recently has technology been available to facilitate the investigation of parallel evolution at various levels of biological organization, which have historically been hidden from view. For example, independently evolved, apparently similar phenotypes might in principle have completely different genetic explanations, suggesting a disconnection between genetic and phenotypic parallelism. Alternatively, the trajectory of adaptation may be severely constrained and highly repeatable across divergent taxa at the level of nucleotide or codon [2,3]. Parallel genetic evolution may occur at the level of nucleotide, gene, or pathway. For example, two lineages may have adapted to similar selection pressures through substitutions in largely non-overlapping genes which nevertheless belong to the same pathway. This would represent convergence at the level of pathway but not the level of gene. Parallel gene expression evolution may occur for transcript abundance, tissue expression, or alternative splicing. In addition to major gaps in our descriptions of the frequency with which parallelism occurs at different levels of biological organization (from single nucleotides to complex phenotypes), we have little understanding of how lineage divergence in biological processes, ecology, or population genetics, may interact to influence the probability of parallelism at different levels of organization. For example, consider populations of two different species evolving in response to a shared, recently changed environment. Because the biology of the two species may differ substantially, the standing variation in the two species may interact with the environmental variation in different ways leading to directional selection on different phenotypes and genes, and this heterogeneity may vary dramatically based on the number of genes and their effect sizes contributing to variation in particular traits within species. Moreover, to the extent that adaptation to novel environments typically results from selection on standing variation, similarities and differences across species in the constellation of segregating variants due to variation in mutation, variation in patterns of mutation-selection balance, or to differences in the magnitude of drift may influence the degree of parallelism. Finally, we have little understanding of how the degree of parallelism scales with relatedness.

While these problems are complex, we set out to begin attacking them in the *Drosophila* model, which has many benefits, including large numbers of species with diverse ecologies [4,5] that can be studied from comparative and population genetic perspectives. Multiple *Drosophila* species show phenotypic latitudinal clines [6–12]. As the central model species for *Drosophila* population genetics, *D*. *melanogaster* latitudinal variation has been subjected to considerable analysis, especially in North American and Australian populations (reviewed in [13]). *D*. *melanogaster* evolved in Africa [14,15]. The species colonized Eurasia on the timescale of thousands of years and colonized the Americas and Australia on the timescale of hundreds of years [15–17]. *D*. *melanogaster* latitudinal clines, are robust, stable on decades long timescales (e.g., Voelker et al. 1978 [18], Hoffmann and Weeks 2007 [11], Eanes 2011 [19]), and often replicated on multiple continents [11,20,21]. More recently, population genomic analyses have been applied to gain a broader picture of the potential influence of spatially varying selection in the species [22–27]. The sibling species, *D*. *simulans*, which is broadly sympatric with *D*. *melanogaster*, has a roughly similar demographic history in that the species evolved in East Africa or Madagascar, and subsequently spread throughout Eurasia, the Americas and Australia [15,16,28,29]. This parallel history has naturally led to the question of whether recent colonization of similar, novel habitats in the two species has been accompanied by similar patterns of latitudinal differentiation. While relatively few studies exist on *D*. *simulans* latitudinal differentiation, the available data suggest that *D*. *simulans* shows weaker latitudinal differentiation at both the phenotypic and genomic levels [7,12,30–33]. This difference between the species has been speculated as being due to a more recent colonization history for *D*. *simulans* [16,29] (so less time for selective differentiation to occur) or due to differences in the ecology and demographics of the two species [34–37]. However, a recent paper on latitudinal gene expression differentiation in both species provided strong evidence for parallel latitudinal adaptation [27]. While additional work will be needed to understand the degree of similarities and differences in latitudinal adaptation in this pair of sister species, here we branch out to highly diverged lineage to continue studying parallel adaptation in *Drosophila*.

*Drosophila hydei* is a member of the *repleta* group of *Drosophila* [38], which shared a common ancestor with the *melanogaster* group roughly 50 million years ago (40–62 mya, [5,39]). The *repleta* group is roughly 20–30 million years old, likely originated in South America [40], and generally exhibits a cactophilic ecology [4,40]. Compared to *D*. *melanogaster*, *D*. *hydei* produces relatively few, very large sperm and exhibits very high re-mating rates [41,42]. *D*. *hydei* is currently cosmopolitan in distribution. Indeed, *D*. *hydei* often appears in massive numbers in the same locations on rotting fruit where Drosophilists typically collect *D*. *melanogaster* and *D*. *simulans* (e.g., Patterson and Wagner 1943 [43]) and is capable of exploiting a wide variety of resources [44]. Thus, while the species retains the ability to exploit cactus as a resource in its ancestral range [40], it is clearly a generalist throughout most, if not all of its current distribution. While the temporal details of the geographic spread of *D*. *hydei* to achieve its current cosmopolitan distribution are currently unclear, Sturtevant in his species description [45] proposed that it first appeared in North America in the late 19^th^ century. Thus, the temporal spread of *D*. *hydei* across North America may be roughly coincident with that of *D*. *melanogaster* [20], suggesting that high temperate regions in North America have been colonized only recently [43], similar to the situation with *D*. *melanogaster*. Given its history, we were interested in understanding whether patterns of latitudinal differentiation in *D*. *hydei* are similar to those in *D*. *melanogaster*. To address this question we produced a reference genome sequence and transcriptome and characterized patterns of sequence variation in high and low latitude populations of *D*. *hydei*. We then compared the properties of *D*. *hydei* genetic variation to the properties of genetic variation from *D*. *melanogaster* populations sampled from the same or similar locations.

**Fig 1:**
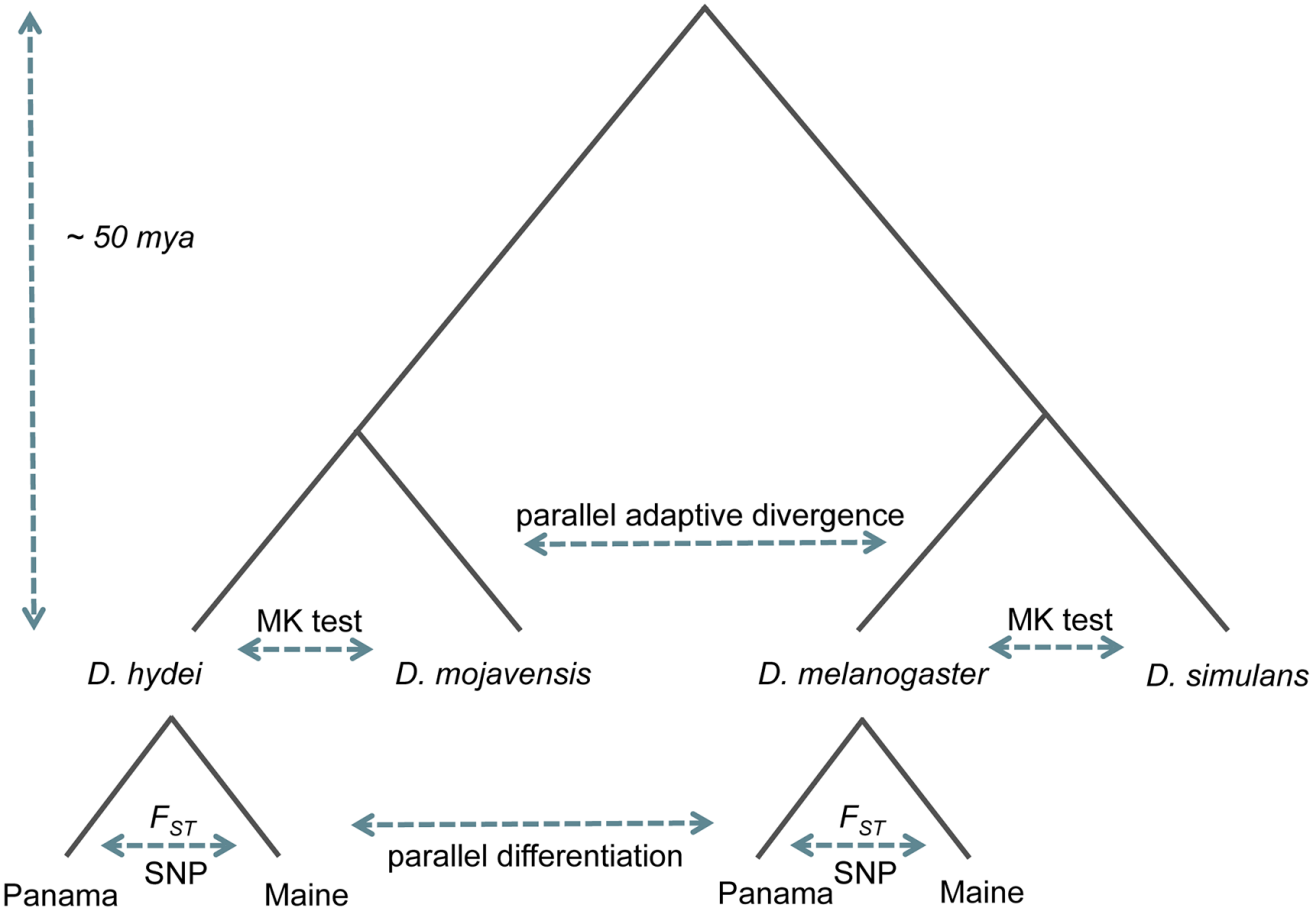
Overview of the system. *D*. *hydei* and *D*. *mojavensis* are members of *repleta* species group, and *D*. *melanogaster* and *D*. *simulans* are members of *melanogaster* subgroup. For each species pair we performed MK tests to identify the targets of parallel recurrent protein adaptation. Maine and Panama population genomes and transcriptomes of *D*. *hydei* and *D*. *melanogaster* were used to study parallel population differentiation between species.

In addition to our interest in parallel latitudinal differentiation in these highly diverged species, we sought to address the question of parallel adaptation at longer timescales that encompass species divergence (Fig 1). A striking conclusion of recent *Drosophila* population genetic work is that a substantial proportion of protein divergence is the result of directional selection [46–49]. This finding, which is based on comparisons of synonymous and non-synonymous variation within and between species [46], has come primarily from investigation of the *melanogaster* subgroup [48,49], though it appears that similar conclusions are likely to hold for the *obscura* group as well [50]. Therefore, in addition to our investigation of geographic differentiation, we used our population genomics data to ask whether there is significant parallelism for the proteins evolving under recurrent directional selection in two highly diverged clades, the *melanogaster* subgroup and the *repleta* group.

## Results

### Genome assembly and gene annotation

We sequenced the *D*. *hydei white* female genome to a high coverage (>170 fold, S1 Table). The genome size estimate based on k-mer frequencies from the short insert library was about 156 Mb (million bp), which is consistent with, though slightly smaller than the species female genome size, 164 Mb, estimated by flow cytometry of ovary nuclei [51]. We used ALLPATHS-LG for the initial assembly and gap filling using Illumina short and long insert reads, and then further filled gaps by SSPACE using corrected PacBio data. After removing bacterial contamination, the final assembled genome was about 139 Mb, with a scaffold N50 of 754 kb and scaffold N90 of 163 kb. The GC ratio of the genome assembly was 39.63%. Analysis of gene content using BUSCO revealed that 95% percent (S2 Table) of the focal genes are included in the assembly, suggesting that the genome is sufficiently well assembled for most population and evolutionary analysis. In total we annotated 14,150 genes (including *ab initio* genes) and 12,380 protein-coding genes (excluding *ab initio* genes). BUSCO and CEGMA analysis (S2 Table) showed that vast majority of conserved genes were well annotated. The assembled *D*. *hydei* genome repetitive sequence composition is comparable to that observed in other *Drosophila* species genome assemblies; 13.31% of the assembled genome is repetitive, including 1.85% retro-elements and 0.5% DNA transposons. Similar to other *Drosophila* species, LTR (long terminal repeats) have the highest abundance, followed by LINEs (long interspersed nuclear elements) [52]. In addition, *Gypsy/DIRS1* has relatively high abundance in *D*. *hydei*, accounting for 0.79% of the genome.

### Muller elements

*D*. *hydei* retains the ancestral *Drosophila* karyotype, which is composed of five major acrocentric chromosome arms (*A*-*E*) plus a dot chromosome (Muller *F*, chromosome *6* for *D*. *hydei*, and chromosome *4* for *D*. *melanogaster*) [53]. Muller elements A-E correspond to chromosome arms *X*, *3*, *5*, *4*, *2*, for *D*. *hydei* and chromosome arms *X*, *2L*, *2R*, *3L*, and *3R* for *D*. *melanogaster* [54]. Using *D*. *mojavensis* and *D*. *melanogaster* synteny we assigned *D*. *hydei* scaffolds to Muller elements A-F (See Methods, S3 Table) based mostly on scaffold gene content. The scaffolds assigned to Muller elements encompass 98% of the assembly (Table 1). We used the scaffold assignments to assign genes to Muller elements/chromosomes (S4 Table). As expected, Muller element assignment results are very similar using *D*. *melanogaster* and *D*. *mojavensis*, since Muller element gene content is generally highly conserved in *Drosophila* [55,56]. However, we observed small differences for Muller element *F* (the dot chromosome) assignment because a number of *D*. *mojavensis* dot chromosome sequences are assembled onto the Muller element E scaffolds (chromosome *3R* for *D*. *melanogaster*, and chromosome *2* for *D*. *hydei*) [56,57]. Because of this, we used the alignment results with *D*. *melanogaster* for downstream analysis of the dot chromosome. A total of 9561 and 2301 genes were assigned to autosomes and *X*-chromosome, respectively. The GC content for the autosomes was 38.8% and the *X*-chromosome content was 40.2%, consistent with previous reports from *D*. *melanogaster* that GC content is greater for the *X* [58].

**Table 1:** Overview of the assembled Muller elements of *D*. *hydei*.

Muller element	Chromosome	Length	Coverage
A	X	27,944,730	59.3
B	3	27,637,053	68.8
C	5	25,365,853	70.6
D	4	24,174,947	71.4
E	2	30,696,216	72.3
F	6	1,068,435	69.1
/	unmapped	3,053,409	/

### Genes and gene families

We first blasted annotated genes against the 20 *Drosophila* species genome annotations [52,59]. Of 12,380 genes included in the analysis, 11,483 had one reciprocal best hit in one of the genomes, which supports previous inferences that current *Drosophila* gene content generally reflects gene content of the *Drosophila* ancestral species [52,59]. We then defined orthologous genes of *D*. *hydei*, *D*. *mojavensis* and *D*. *melanogaster* by using synteny and sequence similarity (reciprocal best hit). This yielded 10,000 putative orthologous genes between *D*. *hydei* and *D*. *mojavensis*, and 9401 such genes between *D*. *hydei* and *D*. *melanogaster*. All downstream orthologous gene related analysis and comparisons are focused on these gene sets. In addition to defining homologous genes and orthologous genes, we also studied gene family number gain and loss using OrthoMCL. Gene copy number appears to be relatively highly conserved with *D*. *mojavensis*; 9298 genes (9105 families) share the same gene copy number as the *D*. *mojavensis* annotation. Genes showing large copy number increases in *D*. *hydei* relative to *D*. *mojavensis* tend to be retro-transposon proteins, such as Tc1-like gene and gag proteins. In total, we found 109 protein-coding genes for which copy number was greater in *D*. *hydei* relative to *D*. *mojavensis*, 41 of which have a homolog in *D*. *melanogaster* (S5 Table). Interestingly, we found duplications of *Ir54a*, *Ir56c*, and *Ir68b*, which are ion-channel genes that are expressed in sensory cilia and may function in detection of chemical stimulus [60]. *CG17387* (testis specific expression, cilium movement) and *SPR* (sex peptide receptor) exhibit species-specific duplications in *D*. *hydei* relative to other sequenced *Drosophila* species [52,59]. In addition, we found *D*. *hydei* duplications of *Apc*/*Apc2*, *fry*, *faf*, *ERR*, *ihog*, *Nox*, *Vps15*, and *Didum*.

### Genomic patterns of nucleotide polymorphism (π)

The overall level of nucleotide heterozygosity in *D*. *hydei* based on 1-kb window means was 0.0019 (Table 2), which is roughly half the nucleotide heterozygosity of North American *D*. *melanogaster* populations [49], and even more severely reduced compared to African *D*. *melanogaster* populations [61,62]. There has been some speculation that in *Drosophila*, genome-wide levels of nucleotide heterozygosity may be determined primarily by the effects of selection on linked sites [48,49,63,64]. This conjecture would predict that all else being equal, species with higher recombination rates would have higher levels of average heterozygosity. *D*. *hydei* euchromatic recombination rates per physical distance are thought to be substantially greater than those of *D*. *melanogaster* [65,66], a conclusion supported by our unpublished estimates of cM/Mb inferred by placing mutants of known genetic location [66] on the assembly. Nevertheless, *D*. *hydei* exhibits substantially lower mean heterozygosity than *D*. *melanogaster*. This difference could result from differences in demographic history or in the intensity of directional selection (though our analysis of adaptive protein divergence below is consistent with roughly equal amounts of protein adaptation in the two species). In any case, the *D*. *hydei* heterozygosity estimates cast some doubt on the proposition that variation in mean heterozygosity across *Drosophila* species will be explained primarily as a consequence of interspecific differences in recombination rates and the interaction of recombination rate variation with selection. We used previously published estimates of *D*. *melanogaster* synonymous heterozygosity for 1-to-1 orthologs [49] and compared them to estimates of synonymous heterozygosity for *D*. *hydei*. The non-synonymous polymorphism and synonymous polymorphism were 0.0010, and 0.0098 respectively, which is smaller than *D*. *melanogaster* homologous genes, 0.0012, and 0.0152 respectively (non-parametric t test, both p <2.2e-16). Thus, the roughly 10-fold greater level of synonymous compared to non-synonymous variation in *D*. *hydei* is similar to that observed in other *Drosophila* species [48,49,67]. If levels of synonymous heterozygosity are determined primarily by selection at linked sites, the extensive chromosome rearrangements that have fixed since the *D*. *melanogaster*-*D*. *hydei* ancestor [68,69] implies that heterogeneous relative recombination rate variation experienced at the scale of genes (or larger) is probably poorly correlated between these species. We observed a very weak but highly significant correlation in synonymous heterozygosity between species (Pearson’s *r* = 0.14, p<2.2e-16), consistent with some degree of conservation for genic parameters of mutation rates and/or selection at *Drosophila* synonymous sites [58,70–74].

**Table 2:** Nucleotide diversity (π) and sequence coverage of each chromosome.

Muller element	Panama	Maine	Coverage_Panama	Coverage_Maine	P-value*
Muller A	0.0020	0.0018	63.48	72.62	< 2.2e-16
Muller B	0.0017	0.0018	57.70	68.69	< 2.2e-16
Muller C	0.0019	0.0020	59.81	70.32	7.01e-11
Muller D	0.0020	0.0021	61.67	70.93	4.71e-08
Muller E	0.0018	0.0019	60.19	70.16	2.14e-14
Muller F	0.0004	0.0004	68.73	77.89	0.9884
Autosome	0.0018	0.0019	59.88	70.07	< 2.2e-16
Genome-wide	0.0018	0.0019	60.86	70.76	< 2.2e-16

*Probabilities for the null hypothesis that nucleotide diversity is the same in the two populations were generated by the Wilcoxon rank sum test on 1-kb windows.

Levels of variation on the *X* chromosome were nearly identical to those observed for the autosomes (Table 2), while the simple neutral equilibrium expectation under equal effective population sizes of males and females is that the *X* will exhibit three-fourths the heterozygosity of the autosomes [75]. Similar observations supporting roughly equal levels of nucleotide heterozygosity on the *X vs*. autosomes have also been made in African population samples of *D*. *melanogaster* and *D*. *simulans* [49,76–78]. In contrast, *X*-to-autosome heterozygosity ratios are substantially less than one in non-African populations of *D*. *melanogaster* (ranging from 0.63 to 0.68 [79] and 0.64 to 0.69 [62]) and *D*. *simulans* [48,80].

We observed a subtle but consistent pattern across windows and Muller elements that a greater proportion of sites were polymorphic in Panama than in Maine (S6 Table). This result, which is robust to variation in quality and coverage, is consistent with the notion that Panama populations are closer to the ancestral geographic distribution of the species and that the recent expansion of *D*. *hydei* to high latitude North American populations [40] has been accompanied by a loss of low frequency variants. However, there is no evidence for a significant bottleneck or serial founder effects, as nucleotide diversity estimated for 1-kb non-overlapping windows was nearly identical in the two populations. Indeed, though a smaller proportion of sites are observed as polymorphic in the Maine sample (S6 Table), Maine generally exhibits slightly greater nucleotide heterozygosity (**π**) compared to Panama (Table 2), presumably as a result of more intermediate frequency variants (S1 Fig). Considering all 1-kb windows in the genome, 42.7% had greater **π** in Panama, 48.2% had greater **π** in Maine, and 9.1% had the same estimated **π** (including windows with no segregating sites in either population) in both populations. The *X* chromosome deviates from this general pattern, exhibiting slightly lower diversity in Maine than in Panama. In the Maine sample, the *X*-to-autosome ratio is 0.94 while in Panama sample the ratio is about 1.09. We investigated the regions showing the greatest difference in **π** between the two populations (1-kb **π** difference > 0.002; 39 genes overlapped these windows (S7 Table). These genes include *nAChRalpha7*, *mxc*, *fz4*, and *X11Lbeta*. Of the 39 genes, 32 (82%) were *X*-linked. This enrichment of *X*-linked genes is not due to a large *X*-chromosome region of geographic differentiation, as these *X*-linked genes are not significantly closer to each other than expected.

### Genomic patterns of differentiation (*F*_*ST*_)

**Fig 2:**
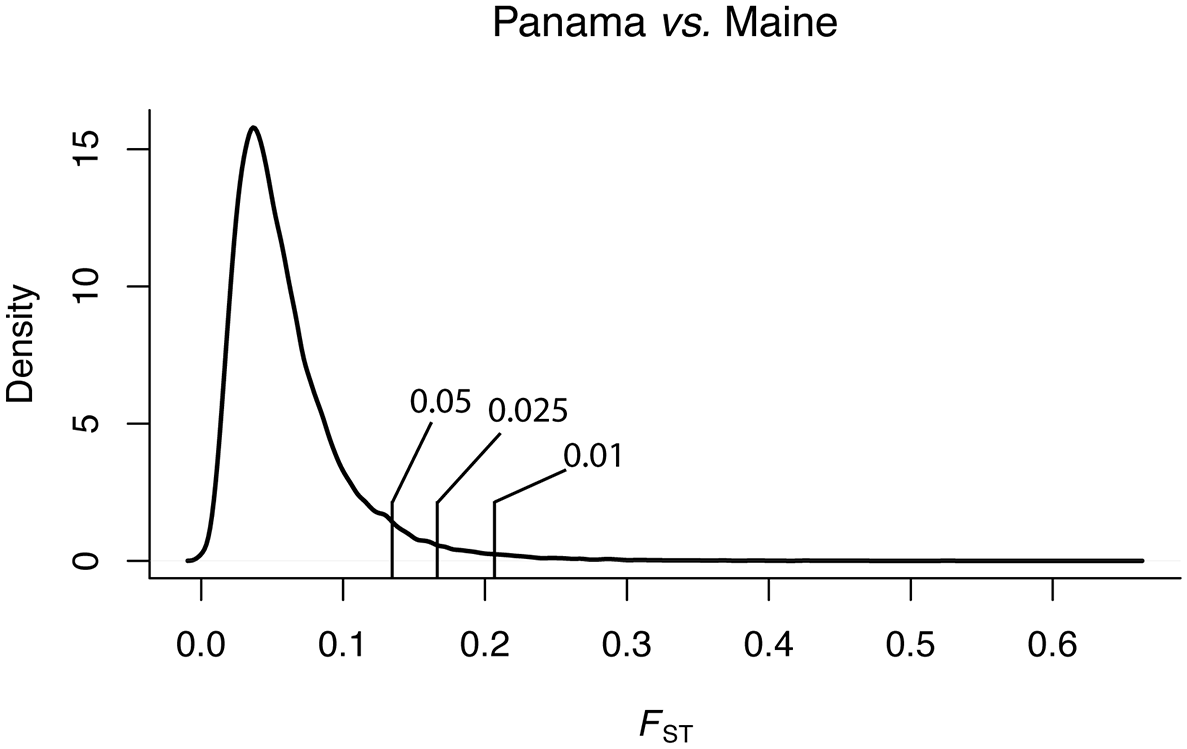
Density of *F*_*ST*_ estimates from 1-kb windows between Panama and Maine populations. The 1%, 2.5%, and 5% tail cutoffs are indicated with hash marks.

Using *F*_*ST*_ estimated in 1-kb non-overlapping windows we identified the windows in the 1%, 2.5% and 5% tails of the distribution. The top 1%, 2.5%, and 5% windows had mean 1-kb *F*_*ST*_ of 0.217, 0.171, and 0.139, respectively (Fig 2, Table 3). These estimates are slightly greater than those observed for outlier 1-kb windows from *D*. *melanogaster* sampled from the same locations (Table 3, 1-kb *F*_*ST*_ non parametric test, Wilcoxon test p < 2.2e-16) [81]. Similarly, median and mean 1-kb *F*_*ST*_ for the *D*. *hydei* genome (Table 3) were 0.050, and 0.061, respectively, which are slightly greater than those of *D*. *melanogaster* populations sampled from the same locations [81]. Similar to observations from US populations of *D*. *simulans* [32,33], levels of geographic differentiation were substantially higher on the *X*-chromosome (1-kb window mean *F*_*ST*_ = 0.077) than on the autosomes (1-kb window mean *F*_*ST*_ = 0.055, Mann-Whitney U, p <2.2e-16, S8 Table), and the pattern remains after coverage correction. Mean *F*_*ST*_ was homogeneous across autosomes (S8 Table). We also characterized *F*_*ST*_ at the level of individual SNPs. As expected, based on the 1-kb window *F*_*ST*_ analysis, SNP *F*_*ST*_ was significantly elevated on the *X* chromosome (p < 2.2e-16, S9 Table).

**Table 3:** Estimates of mean 1-kb *F*_*ST*_ for Maine vs. Panama population samples.

	Mean	SD	1%	2.50%	5%
*D*. *hydei*	0.061	0.042	0.217	0.171	0.139
*D*. *melanogaster*	0.056	0.036	0.193	0.151	0.122

*D*. *melanogaster* estimates were generated from the data of Svetec et al. 2016 using the same parameters described in Methods for *D*. *hydei*.

Shown are the genomic mean; the genomic standard deviation; and the means for windows in the top 1%, 2.5%, and 2.5% of the empirical distribution of 1-kb *F*_*ST*_.

To determine whether genic DNA is over- or under-represented among the most differentiated genomic regions we determined the number of genes overlapping windows in the top 1% of the1-kb *F*_*ST*_ distribution. In total, 201 genes overlapped with these window *F*_*ST*_ outliers. Among them, 123 genes were located on *X*-chromosome. A comparable analysis but with the top 5% 1-kb *F*_*ST*_ outliers resulted in 953 genes, 427 of which were X-linked. For neither cutoff, however, is the number of genes spanned by outlier windows greater than expected based on the proportion of analyzed windows containing genic sequence (p > 0.1). However, because our minimum site and window coverage criteria were quite stringent, there were many more genic than non-genic windows in the analysis, potentially compromising our power to detect genic vs. non-genic enrichments. In addition, we asked if 1-kb windows that overlap genes show greater mean differentiation. We found the mean difference is small (genic window *F*_*ST*_ = 0.059 vs. non-genic window *F*_*ST*_ = 0.063). These results are consistent with previous analyses of latitudinal differentiation in *Drosophila* [23,25] suggesting no major differences in levels of differentiation for genic vs. non-genic regions.

To identify SNPs and genes that may be more likely to experience spatially varying selection we focused on non-synonymous variants. We used a modified Fisher’s exact test to identify separately for each chromosome the outlier non-synonymous SNPs. We found 1070 protein coding genes (S10 Table) having at least one non-synonymous *F*_*ST*_ outlier SNP (FDR 1e-5 and *F*_*ST*_ > 0.15), 308 of which are on X chromosome (p < 2.2e-16). Genes carrying outlier nsSNPs are enriched in Gene Ontology categories such as receptor activity (Benjamini corrected p = 3.18e-04) and molecular transducer activity (Benjamini corrected p = 1.59e-04) (S10 Table). The genes having at least one non-synonymous *F*_*ST*_ outlier SNP in *D*. *melanogaster* were enriched in taste receptor activity (p = 2.90e-04) and related biological processes (S10 Table), suggesting that receptor-related genes may experience spatially varying selection in both species. A number of genes harboring outlier nsSNPs were associated with functions such as regulation of transcription, chromatin modification, cell motion, ovarian follicle cell development, and several additional biological processes. We found that 259 of the 1070 genes overlapped with the top 5% 1-kb *F*_*ST*_ outliers, suggesting that, in agreement with other studies of *Drosophila* [23,25,33], strongly differentiated SNPs tend to be associated with somewhat larger regions of latitudinal differentiation. Of the 259 genes, 122 are *X*-linked, further supporting greater geographic differentiation of the *X* chromosome.

### Parallel evolution of protein-coding genes (nsSNP *F*_*ST*_ outliers)

To identify genes carrying highly differentiated nsSNPs in both *D*. *hydei* and *D*. *melanogaster* we focused on the *F*_*ST*_ outliers identified from the set of “genic” SNPs (Methods) in both species using Fisher’s exact test with midp test correction and estimation of False Discovery Rate (FDR), as described in Svetec et al. 2016 [81]. For both species each chromosome arm was analyzed independently. Among the 9401 one-to-one orthologous genes, 640 *D*. *melanogaster* genes [81] and 1031 *D*. *hydei* genes harbor at least one non-synonymous SNP *F*_*ST*_ outlier (FDR 1e-5 and *F*_*ST*_ > 0.15). Remarkably, we found 110 genes shared between *D*. *hydei* and *D*. *melanogaster*, which represents a 1.57-fold enrichment (hypergeometric test, p = 6.22e-07) compared to the null hypothesis of independence (Table 4, S11 Table). While these shared genes show no major GO enrichment (unsurprisingly given the relatively small number of genes), a number of shared genes were involved in functions such as sensory perception of smell (*scrib*, *Pino*, *Or2a*, and *Ir84a*), detection of chemical stimulus (*Ir94a*, *Ir100a*, *Ir56a*, *Ir40a*, *Ir84a*, and *Ir94c*), and sensory perception of taste (*Gr58b*, *Gr22e*, and *Ir100a*). Several transcription factors, including *pb*, *dpy*, *ush*, *Brf*, and *Elp2* also contain non-synonymous SNP *F*_*ST*_ outliers in both species. Notably, although the *D*. *hydei X* chromosome is enriched for genes carrying nsSNP outliers, there is no enrichment of shared outlier genes on the *X* chromosome (hypergeometric test, p >0.05), probably because there is no over-representation of *X*-linked genes carrying nsSNP outliers in *D*. *melanogaster*. Indeed, shared outliers genes are more likely to be autosomal than *X*-linked (hypergeometric test, p = 1.76e-08). Using the top 400 genes carrying the most differentiated nsSNPs (ranked by FDR) in each species also leads to an observed excess of shared outlier genes (27 shared genes, hypergeometric test, p = 0.01) (Table 4, S11 Table). While a component of the observed excess of shared genes could be attributable to variation in gene-size or SNP density (larger genes are more likely to harbor more SNPs and thus share outliers just by chance), accounting for this source of variation (Methods) also revealed that the observed number of shared genes harboring outlier nsSNPs was significantly greater than expected (78 expected, 110 observed, 1.41-fold enrichment, p = 0.002). We further investigated outlier gene sharing by considering only the 5004 genes that carry at least one nsSNP in both species; this constitutes the set of genes for which we could have, in principle, observed shared outlier genes given the constraints of our data. Of these genes, 513 and 892 carried an outlier nsSNP in *D*. *melanogaster* and *D*. *hydei*, respectively. As was the case in the aforementioned analysis on all genes, the same 110 genes were shared, and the probability that the sharing is due to chance is similar (Table 4, hypergeometric test, p = 0.015). This excess of gene sharing was preserved after accounting for variation in SNP numbers across genes (p = 0.014). These results suggest that despite their long divergence time, distinct biology, and disparate biogeography, that there is a moderate predictability to the patterns of latitudinal differentiation in these two species, at least for genes/protein polymorphisms.

**Table 4:** Number of genes harboring non-synonymous SNP *F*_*ST*_ outliers.

Criteria	Total genes	*D*. *mel* genes	*D*. *hyd* genes	Shared genes	P-value
FDR 1e-5	9401	640	1031	110	6.22e-07
FDR 1e-5, nsSNP in both species	5004	513	892	110	0.015
Top 400 genes	9401	400	400	27	0.01

Total genes are one-to-one orthologous genes between *D*. *melanogaster* and *D*. *hydei* that are used for comparisons. P-values were generated by hypergeometric test.

We further investigated the evidence for parallel responses to spatially varying selection by determining whether outlier nsSNPs in the two species exhibit evidence of more systematic same-direction allele frequency differences (*e*.*g*., in both species the derived allele occurs at higher frequency in Maine) relative to non-outlier nsSNPs. For the outliers nsSNPs located in the 110 shared outlier genes *D*. *melanogaster* showed a marginally significant bias (94 nsSNPs with higher frequency in Maine and 71 with higher frequency in Panama, χ^2^ test, p = 0.05). The comparable *D*. *hydei* analysis revealed a similar trend that was not significant (34 nsSNPs with higher frequency in Maine and 26 with higher frequency in Panama, p = 0.22), though it should be noted that the number of SNPs is small. The probability of the observed trends in both species was relatively small (Fisher’s combined probability = 0.06). We then considered all nsSNPs, not just those in the shared genes, and compared directionality for the top 1000 nsSNPs in each species to the remaining nsSNPs. In this analysis *D*. *hydei* exhibited 537 snSNPs with higher frequency in Maine and 463 nsSNPs with higher frequency in Panama (p = 0.014), consistent with the trend observed in the outlier genes. The comparable analysis for *D*. *melanogaster* revealed 606 nsSNPs with higher frequency in Maine and 394 nsSNPs with higher frequency in Panama (p < 0.001), supporting previous conclusions regarding recent selection in high latitude populations [24,79]. Overall then, there is some support for parallel directionally differentiated nsSNPs, but the effect is not large. However, it is worth noting that the power of these approaches to detect recent, parallel, population-specific allele frequency changes at a set of SNPs enriched for true targets of selection may be compromised by allele frequency changes of nearby neutral SNPs, as well as allele frequency distributions in the ancestral populations and the specific demographic histories of the populations.

### Parallel evolution of gene expression between *D*. *melanogaster*, *D*. *simulans*, and *D*. *hydei*

To investigate whether *D*. *hydei*, *D*. *melanogaster*, and *D*. *simulans* exhibit parallel patterns of latitudinal gene expression differentiation, we performed RNA-seq analysis of *D*. *hydei* Panama and Maine male flies raised at 21°C, and compared those data to comparable existing data from Maine and Panama populations of *D*. *melanogaster* and *D*. *simulans* reared at 21°C [27]. While the lack of *D*. *hydei* biological replicates precluded most formal statistical approaches, given the high coverage of the *D*. *hydei* RNA-seq data and the existing high quality *D*. *melanogaster* and *D*. *simulans* data, we thought the empirical pattern of expression fold-changes between populations was appropriate for generating broad, conservative inferences about gene expression parallelism.

At total of 8760 orthologous genes were expressed in both *D*. *hydei* and *D*. *melanogaster*. We compared the top 300 most differentially expressed orthologous genes for both species and found 25 shared differentially expressed genes, which represents a highly significant excess of shared genes (hypergeometric test, p < 4.4e-04). Different cutoffs (such as the top 500 rather than top 300 genes) returned comparable results. The shared differentially expressed genes include *trp*, *inaF-D*, *ImpL2*, *Eip71CD*, *Cyp6d5*, *Cyp12d1-p*, *Cpr92A*, and *Cpr30F*. These 25 shared genes show no evidence of shared directionality (for example, a gene showed higher or lower expression level in the Panama sample for both the species) (hypergeometric test, p > 0.1). However, the small number of shared genes provides little power to detect such effects. To seek further evidence bearing on the question of shared expression directionality we compared for all genes expressed in both species, the observed fold changes between species for genes showing same direction differences vs. opposite direction differences. We observed that fold changes were slightly more correlated between species for same direction than for opposite direction genes (Pearson’s r abs = 0.60 vs. abs = 0.56, two-tailed test for Fisher’s z-transformations p = 0.03, permutation test correcting gene number, p <0.001). This provides weak, though significant further support for expression parallelism. A total of 5848 orthologous genes were expressed in *D*. *hydei* and *D*. *simulans*. Comparing the top 300 most differentially expressed genes in both species (ranked by fold-change), revealed 25 shared genes (hypergeometric test, p = 0.01). One gene, *Eip71CD*, was differentially expressed in all three species.

While these data support the idea that parallel latitudinal expression differentiation observed between two closely related species, *D*. *melanogaster* and *D*. *simulans* [27] extends to the very distantly related species, *D*. *hydei*, the limitations of our existing expression data leave open the question of the full extent of this form of parallelism.

### Recurrent adaptive protein divergence in *D*. *hydei-D*. *mojavensis and general patterns of parallelism with D*. *melanogaster-D*. *simulans*

We used the McDonald–Kreitman test (MK test) to investigate patterns of adaptive protein divergence between *D*. *hydei* and its close relative, *D*. *mojavensis*. Of the 9315 one-to-one orthologs for which there was sufficient data to carry out an MK test, 807 (8.7%) rejected neutrality at p < 0.05 (S12 Table), while 316 genes (3.4%) had p-values less than 0.01. Of the 807 significant genes, 682 genes deviated from neutrality in the direction of adaptive protein divergence (DoS, Direction of Selection) and had estimated alpha (α = proportion of amino acid fixations explained by directional selection) greater than 0. These results suggest that a substantial proportion of proteins have experienced recurrent directional selection in this clade [47]. In *D*. *melanogaster*, of 9328 genes tested, 1265 (13.56%) had a significant MK test, 593 of which (6.35%) had p < 0.01 (S13 Table). Of these 1265 significant *D*. *melanogaster* genes, 638 (S13 Table) had DoS (Direction of Selection) and proportion of amino acid variants fixed by selection (α) greater than 0, suggesting that a comparable number of genes (682 for *D*. *hydei* vs. 638 for *D*. *melanogaster*) have experienced recurrent adaptive protein evolution in each clade. Thus, the whole genome evidence of pervasive, recurrent adaptive protein divergence in *Drosophila* now includes both the *melanogaster* subgroup [48,49] and the *repleta* group (this report). This conclusion is likely to hold for the *obscura* group as well [50]. A significantly greater proportion of genes that reject the null hypothesis do so in the direction of adaptive divergence for *D*. *hydei* than for *D*. *melanogaster* (χ^2^ test, p < 0.01). The fact that *D*. *melanogaster* is substantially more polymorphic than *D*. *hydei* but exhibits a greater proportion of genes rejecting the null with α < 0 (and comparable to polarized MK tests for *D*. *melanogaster*; Langley et al. 2012 [49]) suggests that a simple explanation of population size variation interacting with slightly deleterious amino acid polymorphisms will not suffice. Note, however, that both *D*. *hydei* (unpolarized MK vs. *D*. *mojavensis*) and *D*. *simulans* (polarized MK [48,49]) exhibit a smaller proportion of genes with α < 0 than *D*. *melanogaster*, and both appear to have higher recombination rates compared to *D*. *melanogaster* [48]. This supports the idea that Hill-Robertson effects associated with recombination rate variation may contribute to the efficacy of selection on new amino acid polymorphisms [82]. However, any model of selection on protein variation must accommodate both estimates of adaptive and deleterious amino acid variation and its interaction with mean recombination rate differences between species and variance in recombination rates within species. For both the *D*. *hydei-D*. *mojavensis* and *D*. *melanogaster-D*. *simulans* clades, the genes showing evidence of recurrent adaptation were enriched on the *X* chromosome (*D*. *melanogaster-D*. *simulans X vs*. autosome is 160 genes vs. 478 genes, *D*. *hydei-D*. *mojavensis* X *vs*. autosome is 133 genes *vs*. 549 genes), supporting faster-*X* adaptation (χ^2^ test, p < 0.001 for both) [48,83]. This is likely a conservative conclusion given that male-biased or male-specific genes, which appear to be more likely then most other classes of genes to experience recurrent protein adaptation (below), are underrepresented on the *X* [47,84–86]. There was no evidence that genes having estimated α < 0 (often interpreted as evidence of deleterious segregating protein variants) are more likely than expected to be shared between clades (hypergeometric test p = 0.18). This is consistent with the idea that divergence in the local recombination rate between these highly diverged species due to extensive karyotype evolution and/or genome-wide differences in recombination rates alters the locus-specific efficacy of selection against deleterious amino acid variants. Alternatively, the distribution of selection coefficients for new amino acid variants may evolve at the gene level.

For the 682 genes having significant MK test with evidence of directional selection in the *D*. *hydei*-*D*. *mojavensis* species pair, 296 showed male-biased or male–specific expression in our reference sequence whole male/whole female transcriptome data. Specifically, 194 genes showed male-specific expression and 102 genes showed male-biased expression. 119 genes showed female-biased gene expression, while no gene showed female-specific expression. Similar to *D*. *melanogaster*, male-biased and male-specific genes were significantly enriched among the genes with evidence of recurrent adaptive protein divergence ((χ^2^ test, p < 0.0001), but female-biased and female-specific genes were not enriched (χ^2^ test, p > 0.1). These results support the idea that male reproduction is a “hotspot” of recurrent protein adaptation. There was no formal GO enrichment for the significant MK genes in *D*. *hydei*-*D*. *mojavensis*.

For the 638 genes significant *D*. *melanogaster* genes, 249 showed male-biased or male–specific expression in our reference sequence whole male/whole female transcriptome data. Specifically, 141 genes showed male-specific expression and 108 genes showed male-biased expression. One significant gene showed female-specific expression, while 120 genes showed female-biased gene expression. Male-biased and male-specific genes were significantly enriched among the genes with evidence of recurrent adaptive divergence ((χ^2^ test, p <0.0001), but female-biased and female-specific genes were not enriched ((χ^2^ test, p > 0.1). GO analysis suggests that genes are enriched in ATP-binding (Benjamini corrected *p* = 0.02) and ubiquitin-protein transferase activity (Benjamini corrected *p* = 0.02). We also found several GO terms including male gamete generation, spermatogenesis, dosage compensation, and regulation of RNA metabolic process that were enriched more than 2-fold but were not significant after multiple testing correction. In general, however, both clades show a strong enrichment of male-related functions for genes exhibiting recurrent adaptive protein divergence.

For *D*. *hydei* we found several dynein proteins among the genes with strong evidence for directional selection (MK test, p < 0.0003, FDR < 0.05). Seven of the top 50 most significant genes (*Dhc98D*, *Dhc16F*, *Dic61B*, *Dhc36c*, *nod*, *Vha100-3* and *Dnah3*) are involved in microtubule-based movement, motor activity and/or ATPase activity, among which, *Dhc98D*, *Dhc16F*, *Dic61B*, *Dhc36c*, and *Dnah3* are components of the axonemal dynein complex. Six of the seven genes show male-biased gene expression or have mutant male fertility phenotypes in *D*. *melanogaster* (FlyBase), suggesting that they may directly function in sperm development and motility. For example, *Dic61B* codes for an axonemal dynein intermediate chain exhibiting strong testis-biased expression (FlyBase); it is required for development and precise assembly of sperm axonemes and is essential for male fertility in *D*. *melanogaster* [87,88]. Given the rapid evolution of sperm length in *D*. *hydei*, along with its close relatives in the *hydei* group, *D*. *bifurca* and *D*. *eohydei* [89], it is tempting to speculate that adaptive evolution of male-specific axonemal dyneins associated with sperm gigantism is related to this phenotype. Also notable among the significant MK genes are five (*aly*, *comr*, *tomb*, *can*, and *sa*) that are homologous to testis meiotic arrest genes in *D*. *melanogaster* (reviewed in White Cooper and Davidson 2011 [90]). These genes are required for regulation of transcripts produced in the primary spermatocyte and whose products function during meiosis and spermatid development. It remains to be seen how the adaptive evolution of these proteins may functionally influence interspecific divergence of gene expression in the primary spermatocytes and how such expression evolution maps onto variation in sperm developmental processes or sperm morphology. GO analysis of the significant MK genes suggests significant enrichment for detection of chemical stimulus (Benjamini corrected p = 2.67e-04) and genes involved in sensory perception of smell (Benjamini corrected p = 1.44e-02).

### Parallel adaptive protein evolution in *repleta* group and *melanogaster* subgroup species

The conclusion that recurrent adaptive protein divergence is common in two highly diverged *Drosophila* clades raises the interesting question of whether the specific proteins exhibiting evidence of recurrent selection in the two clades overlap to a greater degree than expected. For one-to-one orthologous genes, 6578 had sufficient data to perform MK tests in both species pairs. Of these, 467 (7.09%) and 373 (5.67%) genes showed evidence of recurrent adaptive protein divergence in *D*. *melanogaster/D*. *simulans* and *D*. *hydei/D*. *mojavensis*, respectively (S13 and S14 Tables). The two species-pairs share evidence of recurrent adaptive protein divergence in 66 genes, which is highly significant (2.50 fold enrichment, hypergeometric test, p = 1.11e-12, S15 Table). This pattern of excess sharing of significant MK genes holds even when we use a stricter MK test cutoff of p < 0.001. The extensive parallelism supports the idea that there are strong tendencies in *Drosophila* for certain proteins to be frequent targets of recurrent directional selection. The 66 shared genes are dispersed in multiple functional pathways and show no obvious enrichment for particular biological process. However, of the 66 genes, 10 showed male-biased expression while 27 showed male-specific gene expression in *D*. *hydei*; 11 showed male-biased expression while 27 showed male-specific gene expression in *D*. *melanogaster*, supporting the idea that recurrent protein adaption for genes functioning in male reproduction will be a general pattern across the genus *Drosophila*. We used the *D*. *melanogaster* annotation in FlyBase to inspect the biology of these shared genes in slightly greater detail. First, it is worth noting that 35 of the 66 genes have CG numbers but no gene names, which reveals that fundamental biological attributes of many proteins experiencing chronic directional selection in *Drosophila* remain very poorly understood. Of these 35 genes, a large proportion (23 genes in both species and an additional one in *D*. *hydei*) show testis-biased expression and for 12 there is experimental support from proteomics data that the gene product is a component of *D*. *melanogaster* sperm [91]. Turning to the named genes, three (*aly*, *comr*, and *can*) function as regulators of transcription during early spermatogenesis prior to the onset of meiosis. Also showing adaptive protein divergence in both clades is *sneaky*, a sperm acrosome protein required for breakdown of the sperm plasma membrane inside the oocyte [92], and *Dhc98D*, a strongly male-biased axonemal dynein. Also notable is the shared significant gene *qin*, which plays a role in transposon silencing in the female germline [93,94]. The shared gene *mof*, which plays a role in male dosage compensation, supports previous work suggesting that some components of dosage compensation in *Drosophila* are likely to experience frequent directional selection [95], though the possibility that other phenotypes are targets of selection is entirely plausible [96,97].

We examined the estimated α for the 66 shared significant MK genes and found, remarkably, that α was highly correlated across clades (Spearman’s *ρ* = 0.50, p = 1.6e^-5^). This additional form of parallelism implies that beyond the sharing of proteins experiencing recurrent adaptation, for shared proteins the relative contribution of recurrent adaptation to protein divergence tends to be similar across highly diverged clades.

## Discussion

*D*. *hydei* and *D*. *melanogaster* shared a common ancestor several tens of million years ago [5,39] and have highly diverged ecologies, mating systems, and ancestral geographic ranges. While the recent spread of *D*. *hydei* to a cosmopolitan distribution is not as well understood as that of *D*. *melanogaster*, the colonization of high temperate regions in North America by *D*. *hydei* is likely to be recent, similar to the history inferred for *D*. *melanogaster*. Thus, the population genomic analysis of geographic differentiation and of recurrent directional selection on protein sequences in these two species provides some insight into the general repeatability of adaptive evolution on multiple timescales in the *Drosophila* model.

We found, perhaps surprisingly, that parallel latitudinal differentiation at the population genomic level is sufficiently common to be detectable even in our relatively small datasets encompassing only two population samples for each species. Prior to the application of population genomic approaches, *D*. *melanogaster* latitudinal clines had been observed for many phenotypes and genetic variants, which suggested that highly differentiated genomic regions between lower and higher latitude population would be enriched for variants exhibiting clines [22,23], a proposition supported by recent comparison of data from North American cline “endpoints” [81] with data from latitudinal sampling [26]. However, because the existence of latitudinal clines in *D*. *hydei* has not been systematically investigated, we are less confident that strongly differentiated genetic variants, *in general*, are highly enriched for targets of spatially varying selection in this species. Thus, we are limited in our confidence to speculate on the *differences* between these two species in latitudinal differentiation. Nevertheless, the genes showing high levels of latitudinal differentiation in both species provide a glimpse into the prevalence of parallelism and its underlying biological basis. Several of these genes function in detection of chemical stimulus or in taste. The appearance of strongly differentiated DNA repair genes in both species could be related to UV adaptation [81].

One of the major patterns emerging from our population genomic analysis of geographic differentiation is the large *X*-effect. The *D*. *hydei* large *X-*effect is not the result of a small portion of the chromosome showing extreme differentiation, but rather is a general chromosome wide effect. A similar pattern was observed in US *D*. *simulans* [32,33], though not in *D*. *melanogaster* [25]. It remains to be seen through additional comparative work whether *D*. *melanogaster* is highly unusual in this regard and if so, whether selection on autosomal inversions in this species swamps any underlying signal of *X* chromosome dynamics broadly shared across species. A possible demographic explanation for greater *X*-linked differentiation is male-biased dispersal. Because male migrants carry only one *X* chromosome while females carry two, increased male relative to female migration results in a proportional decrease in the number of *X* chromosomes (relative to autosomes) moving from one population to another, which should increase *X* chromosome differentiation [98]. This hypothesis is amenable to both laboratory and field experiments [99–101]. Alternatively, recent models suggest that under a wide range of circumstances the *X* chromosome should show a disproportionate contribution to local adaptation [102]. One might suppose that a chromosome-wide effect should favor the demographic rather than the selective hypothesis. However, the inference from sequence divergence that much of the *Drosophila* genome, including non-coding sequence, is functionally important [103,104] suggests that the selective hypothesis should at least be seriously entertained. Further work will be required to clarify this issue.

While the significant limitations of our population transcriptome data from *D*. *hydei* (relative to our *D*. *melanogaster* and *D*. *simulans* data [27]) weaken our power to detect parallel gene expression differentiation in these species, our results suggest that parallel expression differentiation play a general role in latitudinal adaptation in *Drosophila* [27]. Further quantification of latitudinal gene expression variation in better data from these three species would facilitate the analysis of parallel expression differentiation and permit a more quantitative test of the idea that parallel expression differentiation is significantly more common for closely related species than for more distantly related species, a trend that is consistent with our limited data.

Our analysis of parallel (at the level of the gene) recurrent adaptive protein evolution in two distantly related clades revealed a number of salient results. First, both clades exhibit evidence of rampant adaptive evolution, supporting previous conclusions regarding the prevalence of adaptive protein divergence in *Drosophila* [46–49]. Second, our results suggest that the details of adaptive protein divergence are remarkably similar in these distantly related clades. The two species pairs share many more adaptively evolving proteins than expected under the simple null model. Indeed, it is tempting to speculate that our analysis of shared *repleta* group and *melanogaster* subgroup adaptively evolving proteins has identified a collection of proteins with relatively high probability of evolving adaptively in many *Drosophila* lineages. This conjecture is certainly testable. Third, for the proteins showing evidence of recurrent adaptation in both clades, the proportion of divergence explained by selection is highly correlated. Thus, it appears that there is a surprising level of parallelism in the degree to which protein divergence is determined by directional selection across broad phylogenetic distances in *Drosophila*. The biological patterns of genes with a history of recurrent protein adaption suggest that despite their highly diverged mating systems and reproductive biology, both clades have experienced recurrent protein adaptation at many orthologous genes that are testis-biased, testis-specific, or that are associated with spermatid development and differentiation. Understanding the ultimate cause of this rampant mode of *Drosophila* adaptation remains a substantial challenge. Finally, results from both clades support the notion that adaptive divergence is more common on the *X* chromosome. It is worth noting that the approach used here may substantially underestimate the prevalence of adaptive protein divergence, as MK tests are expected to be underpowered to detect adaptation in small proteins or adaptive protein divergence that occurs in relatively few residues of individual proteins. Whether this bias colors our conclusions about the prevalence of parallel protein adaptation in *Drosophila* remains unclear.

One of the patterns observed here is that there appears to be greater parallelism for long-term adaptive protein divergence, often related to testis expression, than for shorter timescale latitudinal differentiation. This difference could have multiple explanations. First, because these two species are quite diverged they may interact with the environment or with environmental variation in different ways. A corollary of this hypothesis is that the more highly repeatable longer-term parallelism we observed is more likely to involve proteins and pathways experiencing selective processes that tend to be less linked to environmental variation. Male-male interactions, male-female interactions, or genomic conflicts (such as those related to gametic selection or transposable elements) are obvious candidates. Second, to the extent that evolution on short timescales in novel environments may often depend mostly on standing variation, the genetic details of the selection response may differ simply because the constellation of variation available to selection may only be weakly correlated in highly diverged species. Alternatively, if much of the selection response on short timescales depends on alleles ancestrally at mutation-selection equilibrium, then the predictability of differentiation may be reduced by stochastic effects that may dominate even strongly selected low frequency variants, or by evolutionary divergence of the genic parameters of mutation-selection balance.

Finally, it is worth pointing out that we identified three genes, *qin*, *Cht6*, and *Msp-300*, that carry nsSNP latitudinal differentiation outliers in *D*. *melanogaster* and *D*. *hydei* and also show evidence of recurrent adaptive protein divergence between species in the two clades examined here. It remains to be seen whether such potential “hotspots” of adaptation result from agents of selection that tend to be shared on long and short timescales across highly diverged species, or instead, represents a chance occurrence.

## Materials and methods

### Library construction and genome sequencing

#### Reference sequence strain genomic libraries

We obtained a strain carrying a white-eye mutation from the *Drosophila* Species Center (#15085–1641.55, w^1^f^74^) and then inbred it for 8 generations by sib-mating. We selected this strain for its potential future usefulness in transgenic experiments and because reference strain contamination is easily detected. Genomic DNA from females was isolated and used to make 190 bp insert paired-end and 2 kb insert mate pair libraries using the Illumina Truseq kit. These libraries were sequenced on an Illumina HiSeq2000 machine. High quality female DNA from the same strain was used to generate PacBio data by a Pacbio RS Genetic Analyzer using 1X120 min movie.

#### Reference sequence transcriptome

Total RNA was separately extracted from mixed-age male and female adults, made into 190 bp insert paired-end libraries using Illumina Truseq kit, and then sequenced on a HiSeq2000 machine. We used Cuffdiff2 to estimate gene expression (FPKM) with upper quantile normalization and categorized genes with FPKM > 1 as expressed. We categorized a gene as exhibiting sex-biased expression if gene expression was two-fold greater in one sex than the other. Sex-biased genes exhibiting expression level FPKM 0.2 or less for the other sex were categorized as either male- or female-specific.

#### Population genomic sequencing

Flies were collected from Panama City, Panama (PC, collected 01/2012) and Portland, Maine (ME, collected 09/2011), as described previously for *D*. *melanogaster* and *D*. *simulans* [27]. For both populations we sequenced daughters of wild-caught females. One daughter from each of the 28 Panama and 25 Maine wild-caught females were pooled separately to generate a Panama DNA prep and a Maine DNA prep. From each of these two genomic DNAs we prepared a 190 bp insert paired-end library using NEBNext DNA Library Prep Kit (# E6040S), and then sequenced each library on a HiSeq2000 machine.

#### Population transcriptome

The wild-caught females described above were used to establish isofemale lines, from which we sampled flies for RNA-seq experiments. We generated pooled paired-end Illumina libraries (NEBNext DNA Library Prep Kit # E6040S). Twenty isofemale lines from each location were placed on food for 5 days in replicates at both 21°C and 25°C and allowed to lay eggs, after which adults were discarded. One male offspring from each isofemale line was collected, aged for 3 days after emergence (all on the same day), and then pooled prior to RNA isolation to generate an RNA sample from each population. RNA libraries were constructed and sequenced as described above for the reference sequence transcriptomes. The sequencing reads are available under the NCBI BioProject accession number PRJNA373926.

### Estimation of genome size from k-mer analysis

We calculated k-mer frequencies ranging between 13-31mers using Jellyfish [105], and then estimated the genome size using k-mer frequency and coverage [106,107]. In short, the formula is G = Kmer_num/Kmer_depth, where Kmer_num is the total number of k-mers of all the reads and the Kmer_depth is the average depth of k-mers.

### Read quality control, genome and transcriptome assembly

We performed quality control to Illumina short reads, with only high quality reads (Q>30 for each base) being kept for further analysis. PacBio clean reads were first generated from SMRT cell raw data and then further corrected by PacBioToCA [108]. We assembled the reference genome using high quality 190bp insert library reads and 2kb insert library reads by ALLPATHS-LG (release#51298) [109] with standard parameters. We then used corrected PacBio reads to fill scaffold gaps by SSPACE-LongRead [110]. To remove possible microbial contamination we used tblastn to filter contaminated reads. Specifically, all the annotated proteins (see below) were used to blast *Drosophila* species (*Drosophila* 12 species (Clark et al. 2007) and 8 new modENCODE species [59] and *Ensembl* bacteria species by tblastn (-e 1e-5). If more than 1/3 of the total genes on a scaffold had a best-hit map to a bacterium the scaffold was discarded as contamination. Scaffolds that had no annotated genes were used to blast *Drosophila* species and bacteria species by blastn; if such a scaffold had no significant hit to a *Drosophila* species (-e 1e-5) but had a hit (-e 1e-10) to a bacterium, then the scaffold was considered a contaminant.

We used only high quality reads (Q >30, length threshold >30) for transcriptome assembly. Before assembly, we normalized transcripts using normalize_by_kmer_coverage.pl provided by Trinity program (version 2.0.6) using parameter—JM 40G —max_cov 40—pairs_together—PARALLEL_STATS JELLY_CPU 8. Male and female white *D*. *hydei* RNA-seq reads, as well as reads pooled for the two sexes, were assembled using Trinity (version 2.0.6), using parameter—max_memory 40G —min_contig_length 200—CPU 10—inchworm_cpu 10—bflyCPU 10.

### Genome assembly quality evaluation

*Alignment of reads*. To assess assembly quality, high quality Illumina reads from the 190bp paired-end library were aligned to the assembly using BWA (0.7.13, parameter bwa aln -n 0.01 -l 35 -o 1 -d 12 -e 12 -t 8). 94.91% reads could be aligned to the assembled genome, which shows that most reads were incorporated into the assembly. The depth curve plotted based on the alignments showed a unimodal distribution (S2 Fig), suggesting the reads were randomly distributed on the genome and which also suggests that the sequenced strain has very low heterozygosity.

*Core list of genes*. We used two methods to estimate the proportion of highly conserved genes present in the assembly. First, we used BUSCO (Benchmarking Universal Single-Copy Orthologs) [111] to estimate the proportion of the 2765 arthropod orthologous genes that were completely or partially assembled. We also used CEGMA [112] to blast to the genome and identify CEGs (Core Eukaryotic Genes) in the assembly.

### Gene annotation

The MAKER2 genome annotation pipeline was used for gene annotation (maker version 2.31.8, snap version 2013-11-29, hmmer version 3.1b2, TRF version 4.0.9-static, and RepeatMasker version 4.0.5). To improve annotation accuracy we fed the *de novo* assembled transcriptomes, the best translated protein sequences generated by Trinity, and 20 *Drosophila* species protein sequences to help MAKER2 predict gene models, which were then used to train the HMM for *D*. *hydei*. After two rounds of HMM training, MAKER2 was used to predict gene models with *ab-initio* gene prediction algorithms SNAP and Augustus [113]. We generated two annotations, one of which allows *ab-initio* prediction. We used both annotations to estimate the genome quality, but only used the annotation without *ab-initio* prediction for downstream analysis.

### Assigning scaffolds to Muller elements

We aligned annotated *D*. *hydei* genes to the *D*. *mojavensis* and *D*. *melanogaster* genomes using tblastn (-e 1e-10). We assigned a *D*. *hydei* scaffold to a Muller element (A through F) if 55% of annotated genes on a scaffold had the best alignment to one, homologous Muller element based on the blast results to *D*. *melanogaster* and *D*. *mojavensis* [56]. For genes without gene annotation, we blasted sequences to *D*. *mojavensis* genome and used the criteria of minimum 50% alignment length with 30% sequence similarity to determine the Muller element. Using these methods, we assigned 136 of 139 Mb genome sequences to Muller elements A-F.

### Annotation of repetitive sequences and transposable elements

We used TRF (Tandem Repeats Finder, 4.0.9-static) with default parameters to identify non-interspersed repetitive elements. Transposable elements (TEs) were first predicted by homology searches to RepBase TE libraries (version 21.05) using RepeatProteinMask and RepeatMasker (version 4.0.5) with default parameters. We then constructed a *de novo* repeat library using RepeatScout with default parameters and obtained consensus sequences and classification information for each repeat family. Using these RepeatScout consensus sequences as the input library we again searched repetitive elements in the assemblies using RepeatMasker with default parameters. After that, we merged the results from the above pipelines to generate the final classification.

### Mapping of population genomic reads and population genetic analysis

Reads from the Panama City and Maine pools were aligned to the *D*. *hydei* genome using Bowtie2 with the—very-sensitive setting. Variants were called using bcftools (samtools.github.io/bcftools) and PoPoolation2 [114] with a minimal quality score of 30. Following Svetec et al. [81], we required a minimum of 20× coverage at a site in both the Maine and Panama populations and at least two observations of an alternate base call in the entire dataset (two populations) to consider it in the population genetic analysis. We excluded triallelic sites. We calculated expected nucleotide diversity, π, following Kolaczkowski et al. [23] and *F*_*ST*_ following Svetec et al. [81]. For *F*_*ST*_ we performed the odds ratio test for independence using the ormidp.test function in the epitools package in R (medipei.com/epitools/) and then used the p-values from midp tests to calculate the false discovery rate for each chromosome arm using the bioconductor package q-value (http://github.com/jdstorey/qvalue). For scaffolds at least 1-kb long we calculated 1-kb non-overlapping *F*_*ST*_ windows for each chromosome for windows meeting the minimum 20× coverage per site. Windows at the end of a scaffold that were less than 1-kb long were discarded. In total, 99.22% of the assembly was analyzed using 1-kb windows. In addition, for most 1-kb window-based analyses we required that at least 50% of the sites in a window meet our minimum coverage criterion for a window to be included.

For gene-based analyses we included SNPs in the gene region and 1-kb upstream and downstream of the transcript. Within these spans we categorized SNPs as synonymous, non-synonymous SNPs intronic, 3’UTR, 5’UTR, or flanking. To determine whether the number of shared genes with *F*_*ST*_ non-synonymous outliers *in D*. *hydei* and *D*. *melanogaster* was greater than expected, we performed 1000 independent bootstraps to obtain an empirical distribution of shared outlier genes considering the number of SNPs in each gene following Zhao et al. [27], to account for the influence of gene size and SNP number on probability of outlier overlap. To do so, we estimated the number of outlier nsSNP numbers for each of the orthologous genes in *D*. *hydei* and *D*. *melanogaster*, and then randomly picked genes having equal or higher number of nsSNP outliers than the observed genes. We then calculated the number of shared 1-to-1 orthologous genes in *D*. *hydei* and *D*. *melanogaster*. The analysis was repeated 1000 times to generate an empirical distribution of p-values for shared genes harboring nsSNP outliers. We used *D*. *mojavensis* reference to infer the ancestor state of SNPs, and only consider biallelic SNPs, one of which is the same as *D*. *mojavensis* ancestor SNP, for downstream analysis. GO enrichment of each gene list was performed using DAVID v6.8 [115] or Gorilla (http://cbl-gorilla.cs.technion.ac.il).

To determine whether the number of shared genes with *F*_*ST*_ outliers in *D*. *hydei* and *D*. *melanogaster* is influenced by gene size and number of SNPs within genes, we carried out 1000 independent bootstraps to obtain an empirical distribution of shared outlier genes considering the number of SNPs in each gene. We first counted the numbers of outlier nsSNPs in the outlier genes used for comparisons. For example, one set of outlier genes of *D*. *melanogaster* included 369 genes having one nsSNP outlier, 84 genes having two SNP outliers, 27 genes having 3 outliers, etc. We also calculated nsSNP outlier numbers for each gene in the *D*. *hydei* list. We then randomly picked genes that had equal or greater numbers of nsSNPs than the observed nsSNPs in the outlier gene lists in each species, and then calculated the number of shared orthologous genes between *D*. *melanogaster* and *D*. *hydei*. After repeating 1000 times, we obtained the empirical distribution and P-values.

### Population transcriptome sequencing and analysis

Transcriptome sequencing for the samples described above was performed with Illumina RNA sequencing protocols. *De novo* and reference-guided assemblies of high quality clean reads were also performed using Trinity for downstream analysis. The reads were also mapped to the genome using tophat (version 2.0.13). FPKM and differential expression was calculated using Cufflinks and Cuffdiff2, as well as DEseq2 following Zhao et al. 2015 [27]. After generating gene expression and differential expression estimates, we ranked the gene expression fold differences and identified the top 300 differentially expressed orthologous genes. To determine whether there was enrichment for shared latitudinal expression differentiation in *D*. *melanogaster* vs. *D*. *hydei*, as well as *D*. *simulans* vs. *D*. *hydei*, we compared the top 300 most differentially expressed genes in each species and applied the hypergeometric test for independence. We used a χ^2^ test to determine whether genes differentially expressed in both species tend to show greater transcript abundance in either the higher or lower latitude population.

### Gene family evolution

Protein coding genes from *Drosophila* 12 species [52] were downloaded from FlyBase. We used the longest protein sequence of each gene to perform an “all vs. all” alignment using BLASTP (blast+ version 2.2.30+) with e-value cutoff 1e-5. We then use OrthoMCL [116] to cluster genes from different species into gene orthologous groups, following manual check using the blast results. We used reciprocal best hit and synteny relationship (between *D*. *mojavensis* and *D*. *hydei*) to define one-to-one orthologous genes [27]. The reciprocal best hits between *D*. *hydei* and *D*. *melanogaster* as well as *D*. *hydei* and *D*. *simulans* were also used for investigating gene expression differentiation.

### McDonald–Kreitman tests

High quality paired-end reads from the Panama City and Maine libraries were aligned to the genome. We called all bi-allelic SNPs that satisfied the following criteria: 1) minimum mapping quality (Q-score) of 30 [49], 2) minimum coverage of 20 and 3) minor allele called at least 3 times to reduce the possibility that low-frequency slightly deleterious amino acid polymorphisms result in overly conservative conclusions regarding the prevalence of adaptive protein divergence [117,118]. We then used the SNP data to generate alternate reference genomes using an in-house Perl script. Specifically, using each bi-allelic SNP that passed the filtering criteria mentioned above we generated two genomes (a.k.a. alternative references), with each one containing a set of SNPs. We then re-extracted the coding sequence of each gene from alternate references and performed multiple alignments using Genewise to remove insertions and deletions, then re-aligned using PRANK with –codon function for each *D*. *hydei* and *D*. *mojavensis* orthologous gene. To improve statistical power and make our analysis comparable to that from Langley et al. [49], we only carried out MK tests for genes that showed at least one variant in each of four categories, polymorphic, fixed, synonymous, and nonsynonymous. For genes that passed the above criteria we carried out unpolarized McDonald–Kreitman tests using the MK.pl [48,49], using Fisher’s exact test. Significant genes (p < 0.05) were compared to significant genes from comparable unpolarized MK tests for *D*. *melanogaster* (using *D*. *simulans* as outgroup). The *D*. *melanogaster* data included the Raleigh and Malawi samples reported in Langley et al. (2012) [49]. For each gene, we estimated the proportion of adaptive amino acid fixations (α) according to Smith and Eyre-Walker [47], and the Direction of Selection (DoS) index according to Stoletzki and Eyre-Walker [119].

## Supporting information

S1 Fig Histogram of 1-kb π blocks generated from Panama and Maine population. Purple plots are from Panama, and green plots are from Maine. 1-kb π which were larger than 0.01 are not shown here. The average 1-kb π is 0.0018 for Panama and 0.0019 for Maine.
(PDF)

S2 Fig K-mer estimates for reads which were used for *de novo* assembly. The analysis was done using 17, 25, and 31 mers.
(PDF)

S1 Table Sequencing coverage of *D*. *hydei* genome. (DOCX)

S2 Table Assessment of *D*. *hydei* genome assembly and gene annotations. (DOCX)

S3 Table Muller element homology. (DOCX)

S4 Table Total genes on each scaffold and Muller element assignment. (XLSX)

S5 Table Duplicated genes in *D*. *hydei* which have *D*. *melanogaster* orthologous genes. (XLSX)

S6 Table SNP numbers in Panama and Maine. (DOCX)

S7 Table Genes with the most differentiated π and GO analysis. (XLSX)

S8 Table *F*_*ST*_ on each Muller element of *D*. *hydei*. (DOCX)

S9 Table SNP *F*_*ST*_ for each Muller element of *D*. *hydei*. (DOCX)

S10 Table Genes with the most differentiated nsSNP outliers and the GO analysis. (XLSX)

S11 Table Shared genes with non-synonymous *F*_*ST*_ outliers 1) shared genes with non-synonymous *F*_*ST*_ outliers (FDR 1e-5) between *D*. *hydei* and *D*. *melanogaster*. 2) shared top 400 genes with non-synonymous *F*_*ST*_ outliers (FDR 1e-5) between *D*. *hydei* and *D*. *melanogaster*.
(XLSX)

S12 Table *D*. *hydei* genes with significant MK test, and their tissue expression pattern. (XLSX)

S13 Table 1) *D*. *melanogaster* genes with significant MK test, and 2) *D*. *melanogaster* genes exhibiting evidence of adaptive protein evolution. (XLSX)

S14 Table *D*. *hydei* genes exhibiting evidence of adaptive protein evolution. (XLSX)

S15 Table Genes showing evidence of adaptive protein evolution in both the *D*. *melanogaster/D*. *simulans* clade and the *D*. *hydei/D*. *mojavensis* clade. (XLSX)
