# Supplementary figures and images for: Genomics of parallel adaptation at two timescales in Drosophila
Source: PLoS Genet. 2017 Oct 2;13(10):e1007016. doi: 10.1371/journal.pgen.1007016 (PMC5638604; doi:10.1371/journal.pgen.1007016)

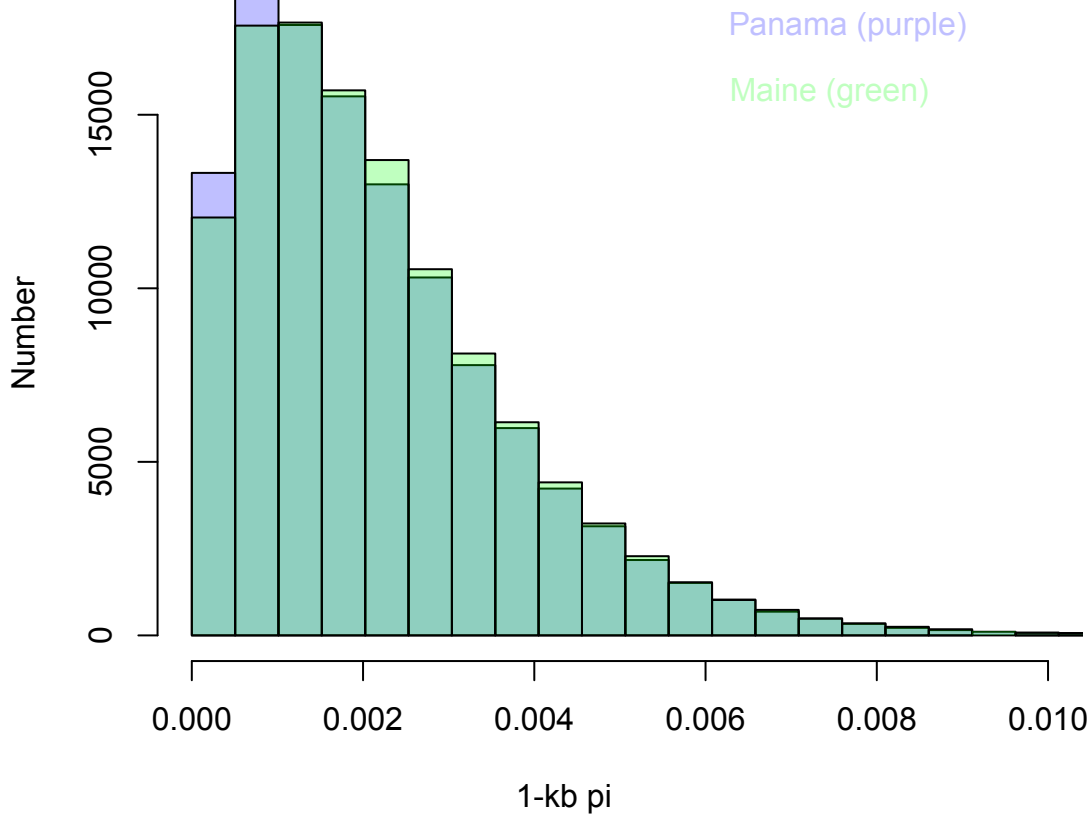

Supplement: S1 Fig — Purple plots are from Panama, and green plots are from Maine. 1-kb π which were larger than 0.01 are not shown here. The average 1-kb π is 0.0018 for Panama and 0.0019 for Maine. (PDF) [file pgen.1007016.s001.pdf]

Number of Kmers

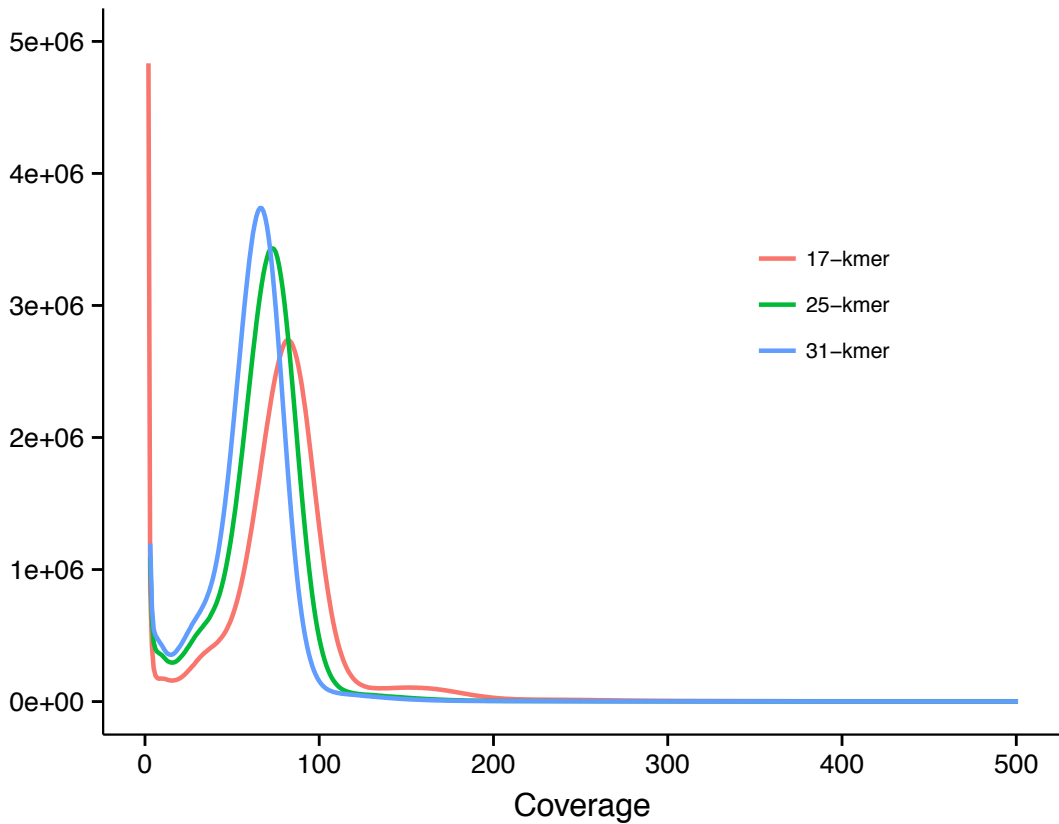

Supplement: S2 Fig — The analysis was done using 17, 25, and 31 mers. (PDF) [file pgen.1007016.s002.pdf]
