# Supplementary material for: Genomics of parallel adaptation at two timescales in Drosophila
Source: PLoS Genet. 2017 Oct 2;13(10):e1007016. doi: 10.1371/journal.pgen.1007016 (PMC5638604; doi:10.1371/journal.pgen.1007016)
Supplement: S1 Table — (DOCX) [file pgen.1007016.s003.docx]

Table S1 Sequencing coverage of *D. hydei* genome.

|  | Technology | Library insert size | Read length | Number of read pairs | % of reads assembled | Data collected (Gb) | Coverage |
| --- | --- | --- | --- | --- | --- | --- | --- |
|  | **Illumina** | female 190bp | 100bp *2 | 89,797,593 | 89.2 | 17.96 | 110.6 |
|  | **Illumina** | female 2000bp | 100bp *2 | 55,860967 | 82.9 | 11.17 | 63.9 |
|  | **PacBio** | 1 x 120 min collection time | 50bp-22kb clean reads | 590,354 | / | 1.48 | 10 |
