# Supplementary material for: Genomics of parallel adaptation at two timescales in Drosophila
Source: PLoS Genet. 2017 Oct 2;13(10):e1007016. doi: 10.1371/journal.pgen.1007016 (PMC5638604; doi:10.1371/journal.pgen.1007016)
Supplement: S2 Table — (DOCX) [file pgen.1007016.s004.docx]

Table S2. Assessment of *D. hydei* genome assembly and gene annotations.

**BUSCO**^1^

|  | Species | Size | BUSCO assessment results |
| --- | --- | --- | --- |
|  | ***D. hydei*** | 139 Mb | Completeness: 95.9% [Duplication: 4.8%]; Fragmented 2.8%; Missing 1.3% |
|  |  | 14150 genes | Completeness: 92.9% [Duplication: 9.7%]; Fragmented 2.8%; Missing 4.3% |
|  |  | 12380 genes | Completeness: 90.3% [Duplication: 8.0%]; Fragmented 2.7%; Missing 6.9% |

^1^ Total number of BUSCO arthropod universal single-copy orthologs is 2675.

**CEGMA**^2^

| **Category** | **#Proteins** | **%Completeness** | **#Total** | **Average** | **%Ortholog** |
| --- | --- | --- | --- | --- | --- |
| Complete | 240 | 96.77 | 306 | 1.27 | 21.67 |
| Partial | 245 | 98.79 | 330 | 1.35 | 26.53 |

^2^ Total number of Core Eukaryotic Genes (CEGs) is 248.

#Proteins = number of 248 ultra-conserved CEGs present in genome; %Completeness = percentage of 248 ultra- conserved CEGs present; Total = total number of CEGs present including putative orthologs; Average = average number of orthologs per CEG; %Ortholog = percentage of detected CEGS that have more than one ortholog. Note that a protein that is deemed to be 'Complete' will also be included in the set of Partial matches, which means in this dataset, 240 genes were complete and 5 were partial.
