# Supplementary material for: Genomics of parallel adaptation at two timescales in Drosophila
Source: PLoS Genet. 2017 Oct 2;13(10):e1007016. doi: 10.1371/journal.pgen.1007016 (PMC5638604; doi:10.1371/journal.pgen.1007016)
Supplement: S3 Table — (DOCX) [file pgen.1007016.s005.docx]

Table S3. Muller element homology.

| Muller element | *D. mojavensis* chromosome | *D. melanogaster* chromosome | D. mojavensis scaffold^1^ |
| --- | --- | --- | --- |
| Muller A | chromosome X | chromosome X | s_6328; s_6308; s_6359; s_6473 |
| Muller B | chromosome 3 | chromosome 2L | s_6500 |
| Muller C | chromosome 5 | chromosome 2R | s_6496 |
| Muller D | chromosome 4 | chromosome 3L | s_6654; s_6680 |
| Muller E | chromosome 2 | chromosome 3R | s_6540 |
| Muller F | chromosome 6 | chromosome X | / |

^1^: result from Schaeffer et al. 2008 genetics.
