# Supplementary material for: Genomics of parallel adaptation at two timescales in Drosophila
Source: PLoS Genet. 2017 Oct 2;13(10):e1007016. doi: 10.1371/journal.pgen.1007016 (PMC5638604; doi:10.1371/journal.pgen.1007016)
Supplement: S6 Table — (DOCX) [file pgen.1007016.s008.docx]

Table S6. SNP numbers in Panama and Maine.

| **Criteria** | **Panama** | **Maine** |
| --- | --- | --- |
| Q20_Cov20_Biallelic | 1115096 | 1039606 |
| Q20_Cov20 | 1136274 | 1061289 |
| Q30_Cov20_Biallelic | 1092018 | 1019971 |
| Q30_Cov20 | 1112153 | 1040603 |
