# Supplementary material for: Genomics of parallel adaptation at two timescales in Drosophila
Source: PLoS Genet. 2017 Oct 2;13(10):e1007016. doi: 10.1371/journal.pgen.1007016 (PMC5638604; doi:10.1371/journal.pgen.1007016)
Supplement: S8 Table — (DOCX) [file pgen.1007016.s010.docx]

Table S8. *F_ST_* on each Muller element of *D. hydei.*

| **Muller element** | **Mean *F_ST_*** | **Sequence coverage** |
| --- | --- | --- |
| Muller A | 0.077 | 68.33 |
| Muller B | 0.056 | 66.08 |
| Muller C | 0.056 | 65.56 |
| Muller D | 0.054 | 65.62 |
| Muller E | 0.056 | 64.01 |
| Muller F | 0.051 | 58.36 |
| Autosome | 0.055 | 65.17 |
| Genome-wide | 0.061 | 65.98 |

Sequence coverage means the average of coverage in Maine and Panama populations.
