# Supplementary material for: Genomics of parallel adaptation at two timescales in Drosophila
Source: PLoS Genet. 2017 Oct 2;13(10):e1007016. doi: 10.1371/journal.pgen.1007016 (PMC5638604; doi:10.1371/journal.pgen.1007016)
Supplement: S9 Table — (DOCX) [file pgen.1007016.s011.docx]

Table S9. SNP *F_ST_* for each Muller element of *D. hydei.*

| **Chromosomes and Muller elements** | **1%** *F_ST_* | **2.5%** *F_ST_* | **5%** *F_ST_* |
| --- | --- | --- | --- |
| Muller A | 0.353 | 0.258 | 0.191 |
| Muller B | 0.260 | 0.192 | 0.144 |
| Muller C | 0.260 | 0.191 | 0.143 |
| Muller D | 0.247 | 0.182 | 0.137 |
| Muller E | 0.259 | 0.190 | 0.142 |
| Muller F | 0.220 | 0.160 | 0.111 |
| Autosome | 0.256 | 0.189 | 0.142 |
| Genome wide | 0.280 | 0.204 | 0.150 |
